# Personalized Recommendations for Physical Activity e-Coaching (OntoRecoModel): Ontological Modeling

**DOI:** 10.2196/33847

**Published:** 2022-06-23

**Authors:** Ayan Chatterjee, Andreas Prinz

**Affiliations:** 1 Department of Information and Communication Technology Center for eHealth University of Agder Grimstad Norway

**Keywords:** descriptive logic, ontology, e-coach, reasoning, recommendation generation

## Abstract

**Background:**

Automatic e-coaching may motivate individuals to lead a healthy lifestyle with early health risk prediction, personalized recommendation generation, and goal evaluation. Multiple studies have reported on uninterrupted and automatic monitoring of behavioral aspects (such as sedentary time, amount, and type of physical activity); however, e-coaching and personalized feedback techniques are still in a nascent stage. Current intelligent coaching strategies are mostly based on the handcrafted string messages that rarely individualize to each user’s needs, context, and preferences. Therefore, more realistic, flexible, practical, sophisticated, and engaging strategies are needed to model personalized recommendations.

**Objective:**

This study aims to design and develop an ontology to model personalized recommendation message intent, components (such as suggestion, feedback, argument, and follow-ups), and contents (such as spatial and temporal content and objects relevant to perform the recommended activities). A reasoning technique will help to discover implied knowledge from the proposed ontology. Furthermore, recommendation messages can be classified into different categories in the proposed ontology.

**Methods:**

The ontology was created using Protégé (version 5.5.0) open-source software. We used the Java-based Jena Framework (version 3.16) to build a semantic web application as a proof of concept, which included Resource Description Framework application programming interface, World Wide Web Consortium Web Ontology Language application programming interface, native tuple database, and SPARQL Protocol and Resource Description Framework Query Language query engine. The HermiT (version 1.4.3.x) ontology reasoner available in Protégé 5.x implemented the logical and structural consistency of the proposed ontology. To verify the proposed ontology model, we simulated data for 8 test cases. The personalized recommendation messages were generated based on the processing of personal activity data in combination with contextual weather data and personal preference data. The developed ontology was processed using a query engine against a rule base to generate personalized recommendations.

**Results:**

The proposed ontology was implemented in automatic activity coaching to generate and deliver meaningful, personalized lifestyle recommendations. The ontology can be visualized using OWLViz and OntoGraf. In addition, we developed an ontology verification module that behaves similar to a rule-based decision support system to analyze the generation and delivery of personalized recommendation messages following a logical structure.

**Conclusions:**

This study led to the creation of a meaningful ontology to generate and model personalized recommendation messages for physical activity coaching.

## Introduction

### Overview

Currently, risk factors associated with unhealthy lifestyle have been recognized as the foremost contributors to chronic illness and mortality in developed countries [1-6]. An e-coach system can guide people and convey the appropriate recommendations in context with sufficient time to prevent and improve living with chronic conditions. It is a set of computerized components that constitute an artificial entity that can observe, reason about, learn from, and predict a user’s behaviors, in context and over time, and engages proactively in an ongoing collaborative conversation with the user to aid planning and promote effective goal striving using persuasive techniques [7-10]. Motivating people toward a healthy lifestyle has been challenging without appropriate and continuous support and correct intervention planning [7-10]. Personalized recommendation technology in health care may be helpful to address such challenges. It requires the proper collection of personal health and wellness data and the right recommendation generation and delivery in a meaningful way. Our previous study [11] focused on creating a meaningful, context-specific holistic ontology to model raw and unstructured observations of personal health and wellness data collected from heterogeneous sources (eg, sensors, interviews, and questionnaires) with semantic metadata and create a compact and logical abstraction for health risk prediction. However, this comprehensive study concentrated on rule-based recommendation generation and semantic modeling of recommendation messages for physical activity coaching.

### Motivation

Generation of motivational messages is essential in e-coaching. Motivational messages provide quick information on time in a more natural and meaningful manner to translate behavioral observations into inspiring, easy-to-follow, and achievable actions. Moreover, these messages must be diverse to make the e-coach system more reasonable and reliable. In activity coaching, personalized motivational messages can offer inspiration for a day, week, or month based on the activity goals. It helps to regain motivation when the individual has lost motivation to attain activity goals. The medium of recommendation delivery can be diverse and depends on personal interaction choices (eg, graphical visualization, pop-up textual notification, and audio-visual material). In existing studies, motivational messages have textual forms that follow a static predefined format; therefore, they are difficult to individualize. Existing ontologies do not include model recommendation message intent, components, and contents important to automatically select accurate messages in e-coaching. Personalized recommendation generation for a healthy lifestyle is closely related to personal preferences. Thus, personal preferences can be of 3 types: activity goal setting (eg, nature of goals—direct vs motivational goals and generic vs personalized goals), response type (eg, mode to communicate extended health state, health state prediction, and customized recommendations for activity coaching), and nature of interaction with the e-coach system (eg, mode, frequency, and medium). In this study, we have gone one step ahead to perform semantic (ontological) modeling of preference data and recommendation messages beyond static textual form to describe its characteristics, metadata, and content information.

The use of ontologies has certain benefits while modeling recommendation messages. It helps to interpret which recommendation message is to be generated using a binary tree-like structure (if-then or if-then-else conditional statement). Interpretability makes identifying the cause-and-effect relationships between data input and data output easy. In ontology, the logical and structural representation of knowledge, hierarchical model structuring (eg, class and subclass model), and inferred knowledge generation with reasoners can solve interpretability problems in decision-making. Furthermore, benefits such as extensibility, flexibility, generality, and decoupling of knowledge help ontology to develop an appropriate solution to model recommendation messages in automatic coaching.

### Aim of the Study

This study proposes a Web Ontology Language (OWL)-based ontology (OntoRecoModel) to deal with personal preferences and recommendation messages and annotate them with semantic metadata information. The *OntoRecoModel* will not only support a logical representation of data and messages but also encourage rule-based decision-making to generate personalized recommendation messages using SPARQL Protocol and Resource Description Framework (RDF) Query Language (SPARQL) as a verification study against different test cases with simulated data. Moreover, we assessed the performance of the ontology against mean reasoning time and query execution time. In *OntoRecoModel*, we annotated the participant’s data with Semantic Web Rule Language (SWRL) and stored the resultant OWL file in a triple-store format for better readability. The *OntoRecoModel* allows automatic knowledge inferencing and efficient knowledge representation to balance a trade-off between complexity, persuasiveness, and reasoning about formal knowledge. The entire study was divided into the following two sections: (1) *OntoRecoModel* design and implementation for semantic annotation and (2) its verification with simulated data. The main contributions of this study were the following:

1. Annotation of personal preferences data (activity goal setting, response type, and interaction type) and recommendation messages in the *OntoRecoModel*.
2. Preparation of semantic rules to execute SPARQL queries for different test cases.
3. Use of the prepared rules to generate personalized activity recommendations.

For this set of semantic data, it will be regarded as an assertion of true facts. The main goal of this paper was to trigger a logical rule of shape (A IMPLIES B) in a logically equivalent manner (NOT [A] or B). If some specific variables are inferred to be true, some suggestions should be provided to the participants of the semantic data source.

### Related Work

This section offers existing knowledge relevant to current research and a qualitative comparison between our proposed ontology and the existing ontologies based on selected categories in Table 1. An ontology is a formal description of knowledge as concepts within a domain and their relationships. It uses existing technologies to develop new ideas through conceptual modeling or proof-of-concept studies to solve general real-world or project-specific semantic modeling problems. There are other approaches to knowledge representation that use formal specifications, such as vocabularies, taxonomies, thesaurus, topic maps, and logical models. However, unlike taxonomy or relational database schemas, ontologies express relationships and allow users to bring together or link multiple concepts in novel ways. Furthermore, all the related ontologies are not available in open source. Therefore, it is not straightforward to make quantitative comparisons between different related studies.

**Table 1:** A qualitative comparison between our proposed study and the existing studies.

Study	Used technologies	Annotation of sensor data	Annotation of personal and health data or health management data	Rule-based recommendation generation	Annotation of preference data	Annotation of recommendation messages
Our study	OWL^a^, HermiT, RDF^b^, SPARQL^c^, TDB^d^, OWLViz, OntoGraf, and Java	Yes	No	Yes	Yes	Yes
Chatterjee et al [11]	OWL, HermiT, RDF, SPARQL, TDB, OWLViz, SSN^e^, SNOMED-CT^f^, OntoGraf, and Java	Yes	Yes	Yes	No	No
Kim et al [12]	OWL	No	Yes	No	No	No
Sojic et al [13]	OWL and SWRL^g^	No	Yes	No	No	No
Kim et al [14]	OWL and FaCT++	No	Yes	No	No	No
Lasierra et al [15]	OWL, RDF, and SPARQL	No	Yes	Yes	No	No
Yao and Kumar [16]	OWL and SWRL	No	Yes	Yes	No	No
Chi et al [17]	OWL and SWRL	No	Yes	Yes	No	No
Rhayem et al [18]	OWL and SWRL	Yes	No	Yes	No	No
Galopin et al [19]	OWL and SWRL	No	Yes	Yes	No	No
Sherimon and Krishnan [20]	OWL and SWRL	No	Yes	Yes	No	No
Hristoskova et al [21]	SOA^h^, Amigo, OWL, and SWRL	No	Yes	Yes	No	No
Riano et al [22]	OWL	No	No	Yes	No	No
Jin and Kim [23]	SSN and IETF YANG	Yes	No	No	No	No
Ganguly et al [24]	OWL	No	No	Yes	No	No
Bouza et al [25]	OWL, Decision Tree, and Java	No	No	Yes	No	No
Villalonga et al [26]	OWL and SPARQL	No	No	Yes	No	Yes

^a^OWL: Web Ontology Language.

^b^RDF: Resource Description Framework.

^c^SPARQL: SPARQL Protocol and RDF Query Language.

^d^TDB: tuple database.

^e^SSN: semantic sensor network.

^f^SNOMED-CT: Systematized Nomenclature of Medicine–Clinical Terms.

^g^SWRL: Semantic Web Rule Language.

^h^SOA: service-oriented architecture.

Kim et al [12] developed an ontology model for obesity management, which realizes spontaneous participation of participants and continuous weight monitoring through the nursing process in the field of mobile devices. The scope of obesity management includes behavioral intervention, dietary advice, and physical activity. Similarly, the study includes evaluation data (BMI, gender, and hip circumference), inferred data to express diagnostic results, evaluation (causes of obesity), success or failure in behavior change, and implementation (education, advice, and intervention). Sojic et al [13] used OWL to model a specific ontology in the obesity field to design reasoning models to personalize health status assessments to be age-specific and gender-specific. The ontology helps to classify personal files according to changes in personal behavior or characteristics over time and automatically infer personal health status, which is of great significance for obesity assessment and prevention. They used SWRL to write the ontology rules. Kim et al [14] proposed a physical activity ontology model to support the interoperability of physical activity data. The ontology was developed in Protégé (version 4.x), and the FaCT++ reasoner verified its structural consistency. On the basis of the automatic calculation paradigm, Monitoring, Analysis, Planning, and Execution, an automatic ontology-based method was developed by Lasierra et al [15] to manage information in the home-based remote monitoring service scenario. Furthermore, they proposed the following three stages [27] for ontology-driven home-based personalized care for the patients with chronic illnesses: stage 1—ontology design and implementation, stage 2—the application of ontology to study the personalization problem, and stage 3—software prototype implementation. The proposed ontology was designed in the Protégé-OWL (version 4.0.2) ontology editor using OWL–Description Logic (OWL-DL) language and verified using the FaCT++ reasoner. Ontology development involves data from heterogeneous sources, such as clinical knowledge, data from medical devices, and patient’s contextual data. Yao and Kumar [16] proposed a new *flexible workflow based on clinical context* method, which used ontology modeling to incorporate flexible and adaptive clinical pathways into clinical decision support system (CDSS). They developed 18 SWRL rules to explain practical knowledge of heart failure. The model was verified using the Pellet Reasoner plug-in for Protégé 3.4. In addition, they developed a proof-of-concept prototype of the proposed method using the Drools framework. Chi et al [17] used OWL and SWRL to construct a dietary consultation system. The knowledge base (KB) involves the interaction of heterogeneous data sources and factors such as patient’s disease stage, physical condition, activity level, food intake, and key nutritional restrictions. Rhayem et al [18] proposed an ontology (HealthIoT)–based system for patient monitoring using sensors, radio frequency identification, and actuators. They claim that the data obtained from medically connected devices are huge, and therefore, lack restraint and comprehensibility and are manipulated by other systems and devices. Therefore, they proposed an ontology model that represents connected medical devices and their data according to semantic rules and, then, used the proposed Internet of Things medical insurance system for model evaluation, which supports decision-making after analyzing the patient’s vital signs. Galopin et al [19] proposed an ontology-based prototype CDSS to manage patients with multiple chronic diseases in accordance with clinical practice guidelines. They prepared a KB based on the clinical practice guidelines and patient observation data. The KB decision rule is based on the *if-then* rule. Sherimon and Krishnan [20] proposed an ontology system (OntoDiabetic) using OWL2 language to support CDSS for patients with cardiovascular disease, diabetic nephropathy, and hypertension to follow clinical guidelines and *if-then* decision rules. Hristoskova et al [21] proposed another ontology-driven environmental intelligence (AmI) framework to support personalized medical detection and alert generation based on the analysis of vital signs collected from patients diagnosed with congestive heart failure. The CDSS system can classify individual congestive heart failure risk stages and notify patients through AmI’s reasoning engine. Riano et al [22] proposed an ontology-based CDSS to monitor and intervene in patients with chronic diseases to prevent critical situations, such as misdiagnosis, undetected comorbidities, lack of information, unobserved related diseases, or prevention. An eHealth system was designed and implemented by Jin and Kim [23] using the IETF YANG ontology based on the semantic sensor network (SSN). This method helped to automatically configure eHealth sensors (responsible for collecting body temperature, blood pressure, electromyography, and galvanic skin response) with the help of information and communication technology and supported querying the sensor network through semantic interoperability for the planned eHealth system. The proposed eHealth system consisted of 3 main components—SSN (eHealth sensor, patient, and URI), internet (eHealth server and KB), and eHealth client (patients and professionals). The proposed semantic model used *YANG to JavaScript Object Notation converter* to convert YANG semantic model data into JavaScript Object Notation semantic model data to achieve semantic interoperability, and then, stored it in a database or KB. Ganguly et al [24] proposed an ontology-based model for managing semantic interoperability issues in diabetic diet management. The development of the framework includes dialogue game rules, DSS with KB (rule library and database), dialogue model based on decision-making mechanism, dialogue game grammar, decision-making mechanism, and translation rules. Bouza et al [25] proposed a domain ontology-based decision tree algorithm and a reasoner to separate instances with more general features for recommender system (*SemTree*) that outperformed comparable approaches in recommendation generation. Chatterjee et al [11] focused on the creation of a meaningful, context-specific ontology (*University of Agder eHealth Ontology [UiAeHo])* to model unintuitive, raw, and unstructured observations of health and wellness data (eg, sensors, interviews, and questionnaires) with semantic metadata and create a compact and logical abstraction for health risk prediction. Villalonga et al [26] proposed a holistic ontology model to annotate and classify motivational messages for physical activity coaching.

**Figure 1:**
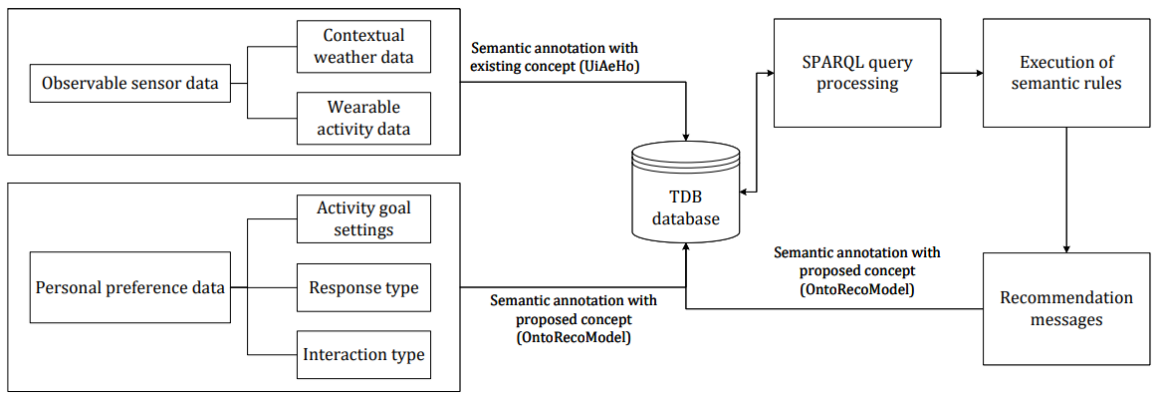
High-level representation of the proposed approach. SPARQL: SPARQL Protocol and Resource Description Framework Query Language; TDB: tuple database; UiAeHo: University of Agder eHealth Ontology.

Most studies have developed ontologies that use OWL to solve data interoperability and knowledge representation problems. However, integrating personal health and wellness data, sensor observations, preference settings, semantic rules, semantic annotations, clinical guidelines, health risk prediction, and personalized recommendation generation remains as a problem in eHealth. We gathered ideas from existing studies to conceptualize our ontology design and implementation. In our previous study [11], we developed *UiAeHo* ontology to annotate personal and person-generated health and wellness data, sensor observations, health status in OWL format, combining SSN and Systematized Nomenclature of Medicine–Clinical Terms. Here, we extended the study to annotate preference settings and activity status and tailored recommendation messages for activity e-coaching. The design and development of *UiAeHo* were focused more on obesity and overweight case studies However, this study focuses strictly on activity coaching and recommendation modeling. In addition, our proposed ontology was verified with semantic rules to generate different categories of recommendation messages for different cases. The high-level graphical representation of the proposed approach has been depicted in Figure 1 to show a distinction between *OntoRecoModel* and *UiAeHo* ontologies. *OntoRecoModel* annotates the following 3 types of data: sensor data (activity and weather), personal preference data, and personalized recommendations. Annotation of the sensor data in *OntoRecoModel* was based on the existing *UiAeHo* ontology following a semantic structure. Sensor data (activity data and contextual weather data) were included in this ontology design to exhibit that our *OntoRecoModel* can generate contextual and personalized recommendations in combination with personal preference data and semantic rules.

## Methods

### Domain Ontology

Ontology supports flexibility in its design to solve real-world modeling and knowledge representation problems. It is a formal model of a specific domain, with the following essential elements: individuals or objects, classes, attributes, relationships, and axioms. The class diagram of a program written using object-oriented programming [28,29] visually depicts an ontology. The concept of ontology was created thousands of years ago in the philosophical domain, and it has the design flexibility of using existing ontology [29,30].

The open-world assumption knowledge representation style uses OWL, RDF, and RDF schema syntax. It can be optimized using the ontology model, and the consistency of its logic and structure can be verified using the ontology reasoning machine. An ontology О is defined as a tuple Ω=(Ć, R), where Ć is the set of concepts and R is a set of relations. An ontology has a tree-like hierarchical structure (О_h_) with the following properties [31,32]:

1. L=levels (О_h_)=total number of levels in the ontology hierarchy, 0≤n≤L, where n 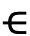 Z^+^ and n=0 represent the root node
2. C_n,j_=a model classifying О at a level n; where, j 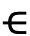 (0, 1,....., |C_n_|)
3. |C|=number of instances classified as class C
4. E=edge (C_n,j_, C_n-1, k_)=edge between node C_n,j_ and its parent node C_n-1, k_

### Ontology Design Approach

An ontology can be designed in 5 ways: inspirational, inductive, synthetic, deductive, and collaborative [33]. We used a mixed method in our ontology design after combining the inspirational and deductive approaches. The inspirational approach helped us to identify the need for the ontology design, and the deductive approach focused more on the development of the *OntoRecoModel* model in Protégé. Moreover, the deductive approach helped us to adapt and adjust general principles to develop an anticipatory ontology of personalized activity recommendations as a study case. It includes general concepts that are filtered and refined to personalize specific domain subsets. The overall approaches were distributed in the following phases:

1. Literature search: we identified the necessary ontology components in healthy lifestyle management through a literature review, as described in the *Related Work* section. This study aimed to integrate ideas from the related ontology development in our proposed work.
2. Ideation: we discussed with 12 experts in the domain of information and communication technologies with research background in health care to design the concept of the ontology to fit in an activity e-coaching.
3. Annotation: we designed and developed the *OntoRecoModel* ontology to annotate personal preference data and motivational recommendation messages.
4. Rule base: we created a rule base for SPARQL query engine for query execution and personalized recommendation message generation (rule-based inference).
5. Verification: we verified our proposed *OntoRecoModel* ontology using simulated data against different test cases.

The feasibility study of the proposed *OntoRecoModel* consists of the following steps—(1) designing the ontology to fit in activity e-coaching concept; (2) modeling the ontology in the Protégé open-source platform and reasoning with HermiT reasoner; (3) integrating the concepts, such as annotation of personal preference data and motivational recommendation messages in *OntoRecoModel*; (4) implementing *OntoRecoModel* with logical axioms, declaration axioms, classes, instances, object properties, and data properties; and (5) setting up the rule base for ontology verification with SPARQL queries. We further discussed how interpretation can be associated with rule-based activity recommendation generation.

The specifications related to this study, as maintained by World Wide Web Consortium, are XML, URI, RDF, Turtle, RDF schema, OWL, SPARQL, and SWRL. The following terms are related to *OntoRecoModel* representation and processing:

1. Propositional variables (the atomic name of the truth value can be changed from one model to another)
2. Constants (the only propositional variables are TRUE and FALSE; thus, their truth values cannot be changed)
3. Operators (a set of logical connectors in each logic)

Here, we used operators, such as NOT, AND, OR, IMPLIES, EQUIV, and quantifiers (a set of logical quantifiers in a given logic). In this study, we used FORALL as the universal quantifier, EXISTS as the existential quantifier, quantification clause (a set of propositional variables connected by operators and quantifiers), clause (a quantification clause without any quantifier), formulas (a collection of clauses and quantified clauses linked together by logical operators), and process models (a collection of assignments for each propositional variable, so that when simplified, the process will lead to the constant TRUE).

Different open-access ontology editors are available in the market, such as NeOn Toolkit, Protégé, FOAF editor, TopBraid Composer, WebOnto Ontolingua Server, OntoEdit, WebODE, and Ontosaurus. The editors support the development of OWL-based ontologies. In addition, these editors support reasoning. The reasoner is a crucial component for using OWL ontology [11]. It derives new truths about the concepts that are modeled using OWL ontology. All queries on OWL ontology (and its imported closures) can be performed using reasoners [11,34,35]. Therefore, the knowledge in the ontology may not be explicit, and a reasoner is needed to infer the implicit knowledge to obtain the correct query results. If reasoner implementation is needed, the reasoner must be accessed through application programming interface (API). The OWL API includes various interfaces for accessing OWL reasoners. Reasoners can be categorized into 3 groups—OWL-DL, OWL–expression language, and OWL–query language [11,34-42]. This study considered Protégé (version 5.x) as an ontology editor for ontology design and development, OWLViz for ontology visualization, and HermiT (version 1.4.x; ∈OWL-DL) reasoner for validating the ontology structure. In addition, we used an open-source Apache Jena Fuseki server [39] for SPARQL processing [43,44] with a tuple database (TDB). TDB supports Jena APIs [45,46] and can be used as a stand-alone high-performance RDF storage.

### Ontology Modeling

Ontology modeling in Protégé can be classified into the following 2 categories: OWL-based and frame-based categories. We have used Protégé-OWL editor to model *OntoRecoModel* following the open KB connectivity protocol using classes, instances (objects), properties (object properties and data properties), and relationships. The steps of *OntoRecoModel* modeling in Protégé are described in Textbox 1.

**OntoRecoModel modeling steps in Protégé.**

**Step 1**

Creation of a new Web Ontology Language project in Protégé and save it as a Turtle Resource Description Framework (RDF) format (OntoRecoModel.ttl)

**Step 2**

Create named classes under the superclass *owl:Thing,* maintaining consistency

- Create a group of classes (G=[C_1_, C_2_,......, C_n_])
- Define disjoint classes (Cx ∩ Cy=[ø], where Cx and Cy 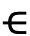 G)
- Define subclasses
- Define disjoint subclasses

**Step 3**

Creation of Web Ontology Language properties after identifying classes and their properties

- Object properties (association between objects)
- Data properties (relates objects to XML schema datatype or rdf:literal)
- Annotation properties to annotate classes, objects, and properties

**Step 4**

Define nature of the properties

- Subproperties (A ⊆ B, where A and B are two nonempty sets)
- Inverse properties (x×y=I, where x, y
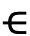
A; I=identity element)
- Functional properties (X=A×X, where X is the set of all sequences <a1, a2,..., an> for a1, a2,.., an ⋲ A)
- Inverse functional properties (for a function f: X → Y, its inverse f^-1^: Y → X, where X, Y 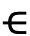 R)
- Transitive properties (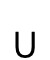
S ⊆ S or if x=y and y=z, then x=z, where x, y, z ⊆ S set)
- Symmetric properties (if x=y, then y=x, where x, y ⊆ S set)
- Reflexive properties (x=x, where x ⋲ R)

**Step 5**

Addition of existing ontology classes (eg, semantic sensor network ontology classes to annotate sensor observations)

**Step 6**

Define property domain (D) and range (R) for both object properties and data properties as axioms in reasoning

**Step 7**

Define property restrictions

- Qualifier restrictions (existential and universal)
- Cardinality restrictions (≥1)
- hasValue restrictions (datatype)

**Step 8**

Ontology processing with reasoner to check structural and logical consistency and compute the inferred ontology class hierarchy

- Blue color class in inferred hierarchy for reclassification
- Red color class in inferred hierarchy for inconsistent class

**Step 9**

Remove inconsistencies from the ontology tree using pruning method

**Step 10**

Query processing with SPARQL Protocol and RDF Query Language and storing the *Terse RDF Triple Language* file into tuple database for persistence

### Ontology Implementation

#### Scope

We have planned to integrate the proposed *OntoRecoModel* model into an automatic activity coaching system for the semantic representation of activity sensor data, weather sensor data, personal preference data, and recommendation messages. The annotation of sensor data was pre-existing, and we used the concept from our previous study [11]. Furthermore, we showed a direction to use the proposed ontology model for automatic rule-based tailored activity recommendation generation with SPARQL queries to motivate individuals to maintain a healthy lifestyle. *OntoRecoModel* has gone one step forward to represent motivational recommendation messages beyond the *string* representation. Furthermore, the rule base helped to interpret the logic behind recommendation generation with logical AND and OR operations. We verified the ontology against a few test cases, which consisted of simulated data.

**Figure 2:**
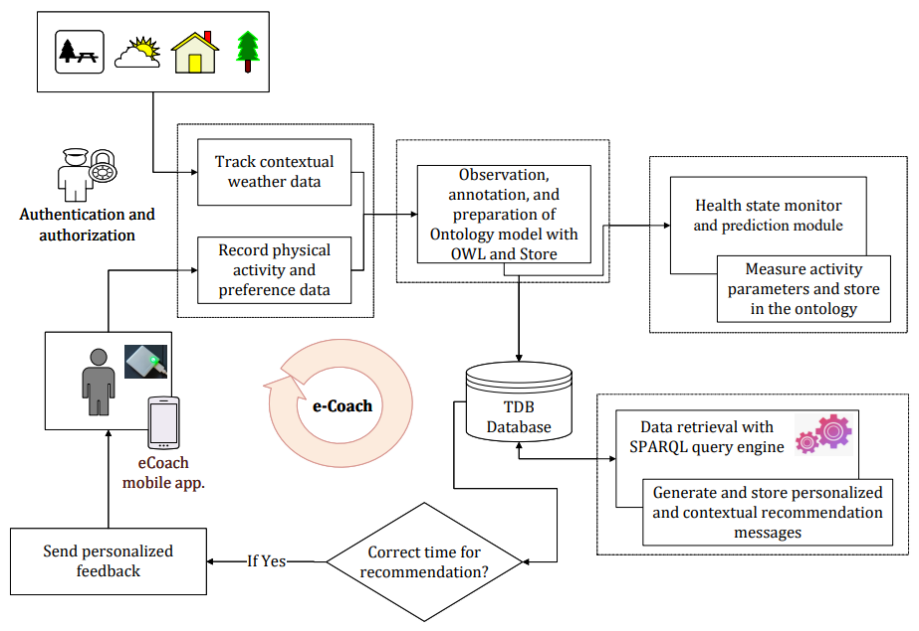
The modules of the e-coach prototype system. OWL: Web Ontology Language; SPARQL: SPARQL Protocol and Resource Description Framework Query Language; TDB: tuple database.

The targeted activity e-coach system has three modules, as depicted in Figure 2—(1) data collection and annotation module, (2) health state monitor and prediction module, and (3) recommendation generation module. In the data collection and annotation module, we showed a direction to annotate personal preference data essential for personalized recommendation generation. Health state monitor and prediction models periodically load individual activity data and analyze them using a data-driven machine learning (ML) approach or a rule-driven binary conditional approach. We considered a rule-driven approach for monitoring individual activity data using SPARQL queries. It determines whether a participant is sedentary or active over a day based on the recorded activity data. The annotated query processing results are stored in the database. Then, the personalized recommendation generation module combines the annotated SPARQL query results with the annotated preference data to generate tailored recommendation messages for motivation, which may help individuals to achieve their activity goals.

#### Annotation of Sensor Data

As shown in our previous study, this study achieved annotation of activity sensor data and contextual weather sensor data using pre-existing SSN ontology [11]. We used a similar logic; however, we annotated them more realistically. We examined the recorded activity parameters of different wearable activity sensors, such as Fitbit Versa, MOX2-5, and Garmin, and discovered that the following parameters are essential and common across these activity sensors: sedentary time, low physical activity (LPA) time, medium physical activity (MPA) time, vigorous physical activity (VPA) time, and total number of steps. Therefore, in this ontology, we annotated these activity parameters. Similarly, we analyzed data from different weather APIs, such as AccuWeather, Yr.no, and OpenWeather API. We found that the following observable weather parameters are common across these APIs: city, country, weather code, status, description, temperature, real feel, air pressure, humidity, visibility, and wind speed. Thus, it may help *OntoRecoModel* to be functional, irrespective of the choice of standard activity sensor and weather APIs.

#### Annotation of Personal Preference Data

Personal preferences reflect individual expectations from an e-coach system. We planned to collect personal preference data at the beginning of the individual e-coaching session. We classified preference data into three categories: (1) activity goal settings, (2) response type for coaching, and (3) interaction type. Activity goals were categorized into 2 groups: personalized versus generic and direct versus motivational. The generic goals in activity coaching are the general activity guidelines set by the World Health Organization [47]. Personalized activity goals can be of multiple types (eg, weight reduction, staying active, body fat level, and proper sleeping). Direct goals tell the participant to perform direct activities (such as walking 2 km tomorrow).

In contrast, motivational goals inspire the participants to perform some tasks through persuasion (eg, If you walk 1 km further, you can watch an excellent soccer game). Response type for e-coaching can be either direct (eg, a pop-up message or notification to receive activity progression alert) or indirect (eg, graphical representation of activity progression). Individuals can be encouraged with personalized, evidence-based, and contextual response generation and its purposeful presentation (eg, graphic illustration, selection of colors, contrasts, visual aspects of movements, and menus, which are adjustable with device type). Interaction is an action that occurs owing to the mutual effect of ≥2 objects. The concept of 2-way effects is essential in interaction, not 1-way causal effects. The interaction types can be the mode (eg, style and graph), medium (eg, audio, voice, and text), and frequency (eg, hourly, daily, weekly, and monthly). Notification generation is a subcategory of interaction and may be persistent or nonpersistent.

#### Annotation of Recommendation Messages

The recommender module generates personalized and contextual recommendations based on the prediction status. The recommendations can be direct (eg, pop-up notifications as alerts) or indirect (eg, visual representation). Direct or immediate notifications can contain 2 types of messages: to-do or formal (eg, You need to complete 1500 more steps in the next 2 hours to reach your daily goal) and informal (eg, Good work, keep it up! You have achieved the targeted steps). Therefore, we broke down the recommendation message concepts into intents and components. Intent defines the message’s intention (eg, formal or informal). Message components define time, element (eg, data types in XML schema definition language), action (eg, pop-up and graphical visualization), and subject. An individual can receive >1 meaningful recommendation message based on the one-to-many relationship.

### Ontology Classes and Properties

**Figure 3:**
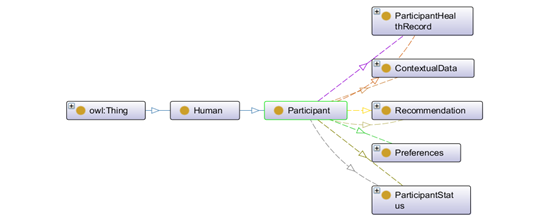
High-level graphical representation of participant using OntoGraf in Protégé. OWL: Web Ontology Language.

**Figure 4:**
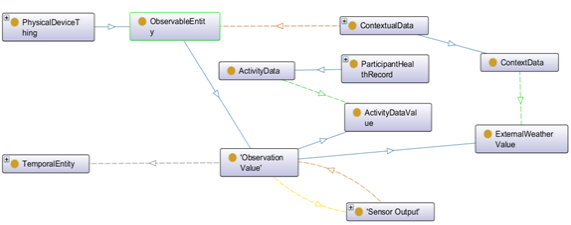
High-level graphical representation of observable data using OntoGraf in Protégé.

**Figure 5:**
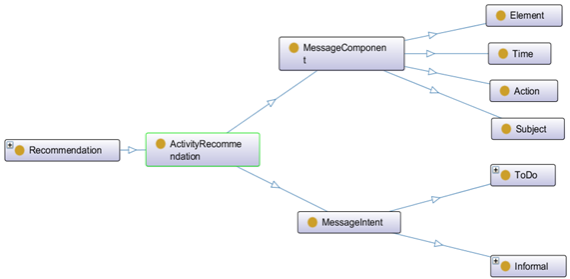
High-level graphical representation of recommendation using OntoGraf in Protégé.

**Figure 6:**
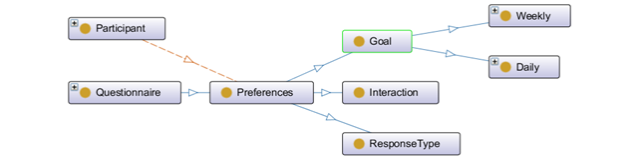
High-level graphical representation of preferences using OntoGraf in Protégé.

Figure 3 to 6 describe *OntoRecoModel* with mandatory classes to annotate the sensor, preference, and recommendation data.

Participant is the subclass of the human class (Figure 3). They have dedicated role and credentials (objectProperties: hasRole, hasPassword, and hasUniqueUserId) to authorize and authenticate themselves in the system. Participants are adults (both men and women), digitally literate, and clinically fit individuals. They are associated with the data properties such as hasAge, hasDesignation, hasEmail, hasFirstName, hasLastName, hasGender, and hasMobile. Each participant has their health record (hasHealthRecord), such as activity data; status (hasStatus), such as *active* or *inactive*; context information, such as weather status; preferences (hasPreferences); and recommendations (hasReceivedRecommendation).

Sensor data are ObservableEntity (Figure 4). Observation value is the subclass of ObservableEntity. ActivityDataValue and ExternalWeatherValue are the subclass of Observation value class. ActivityData and ActivityDataValue are linked to represent individual activity data. ActivityData class is a subclass of ParticipantHealthRecord and has objectProperty—hasBeenCollectedBy to represent associated activity data values (class: ActivityDataValue) as an observable entity. We have planned to collect activity data (such as steps, LPA, MPA, VPA, sleep time, and sedentary bouts) with a wearable MOX2-5 activity sensor. In contrast, contextual data are observable weather-related data (city, country, weather code, status, description, temperature, real feel, air pressure, humidity, visibility, and wind speed), which are planned to be collected through the OpenWeather web interfaces. ContextData class is the subclass of ContextualData class and linked with ExternalWeatherValue to represent contextual weather data. TemporalEntity class represents the time stamp when the observational data have been captured and personalized recommendations have been generated (data property: hasDateTime).

Recommendation is a broad area, and we considered only activity recommendations in this study. ActivityRecommendation is a subclass of Recommendation class and parent to the MessageIntent and MessageComponent with the following objectProperties: hasMessageIntent and hasMessageComponent. MessageIntent class is the parent to ToDo and Informal classes with the following objectProperties: hasRecoInformal and hasRecoToDo (Figure 5). MessageComponent is the parent of Time, Element, Action, and Subject classes with the following objectProperties: hasTime, hasElement, hasAction, and hasSubject. Preferences is a subclass of the Qualifier class and related to the Goal, Interaction, and ResponseType (subclasses of the Preference class) with the following objectProperties: hasInteractionType, hasResponseType, and hasGoal. Preference class is a questionnaire-based method to receive participant’s choices on goal setting, response type for e-coaching, and nature of interaction with the e-coach system.

Preference class has 3 subclasses: ResponseType, Goal, and Interaction. Goal class has 2 subclasses: Daily and Weekly (Figure 6). Each activity recommendations are either *generic* or *personalized*. Thus, recommendation generation depends on the assessment of the health status of the participants, regarding activity measurement and contextual information. Contextual data help recommend participants to plan indoor or outdoor activities based on external weather conditions. Table S1 in Multimedia Appendix 1 [48-51] summarizes the set of identified recommendation messages used for the test setup (ontology verification) and prepared based on positive psychology [52] and the concept of persuasion [48]. Recommendations generated on day *n* will reflect daily activity and contemplate what to perform on the day *n+1* to achieve the weekly goal. Preference data are personalized and customizable. All the necessary data for this study and their nature are summarized in Table S2 in Multimedia Appendix 2.

Description logic is the formal knowledge representation of ontology language, which provides a good trade-off between the expressiveness, complexity, and efficiency of knowledge representation and structured knowledge reasoning. We have the following proposition variables and recommended messages with their links to ensure that the paper is fully understood. Now, we need a set of clauses so that specific models can assign these variables to true, which triggers the sending of recommendations. SROIQ Description Logic [53] is the logic that provides the formal basis for OWL2 and has been used as the formal logic for reasoning in this study (Table S3 in Multimedia Appendix 3).

### Ontology Verification

#### Test Cases With Simulated Data

We considered 8 test cases, as described in Table S4 in Multimedia Appendix 4, with simulated data for the proposed ontology verification. In the table, all the data are simulated. Therefore, no ethical approval was required. Cases 1 to 4 were associated with goal type—*generic* (World Health Organization standard guidelines to stay active for an entire week). Cases 5 to 8 were associated with goal type—*personalized*. More detailed description of different cases is provided in Textbox 2. The primary objective of the test cases was to check whether the daily step goal and daily sleep goal were achieved. The sedentary time and total time of VPA, MPA, and LPA were evaluated as a part of the secondary goal achievement. Daily goal achievement consisted of both primary objective and secondary objectives.

For all the test cases, the contextual weather data were considered constant (Table S5 in Multimedia Appendix 5). These test cases were added to the proposed ontology as individuals. SPARQL query processor engine processed the simulated data against certain test cases.

**Different test cases and their description.**

**Goal type: *Generic***

- Case 1 (11): Daily step goal and sleep goal are achieved.
- Case 2 (10): Daily step goal is achieved; however, sleep goal is not achieved.
- Case 3 (01): Daily step goal is not achieved; however, sleep goal is achieved.
- Case 4 (00): Daily step goal and sleep goal are not achieved.

**Goal type: *Personalized***

- Case 5 (11): Daily step goal and sleep goal are achieved.
- Case 6 (10): Daily step goal is achieved; however, sleep goal is not achieved.
- Case 7 (01): Daily step goal is not achieved; however, sleep goal is achieved.
- Case 8 (00): Daily step goal and sleep goal are not achieved.

**Note:**

- 1 and 0 are two binary numbers and represent an on-off switch.
- 0 indicates that certain feature is false and 1 indicates that certain feature is true.
- Their combination (00, 01, 10, and 11) represents the following 2 combined features: daily step goal and daily sleep goal.
- The combination produces a total of 2^n^ possible test cases (00, 01, 10, and 11) for each goal type.

#### Rule Creation for SPARQL and Rule Execution

**Figure 7:**
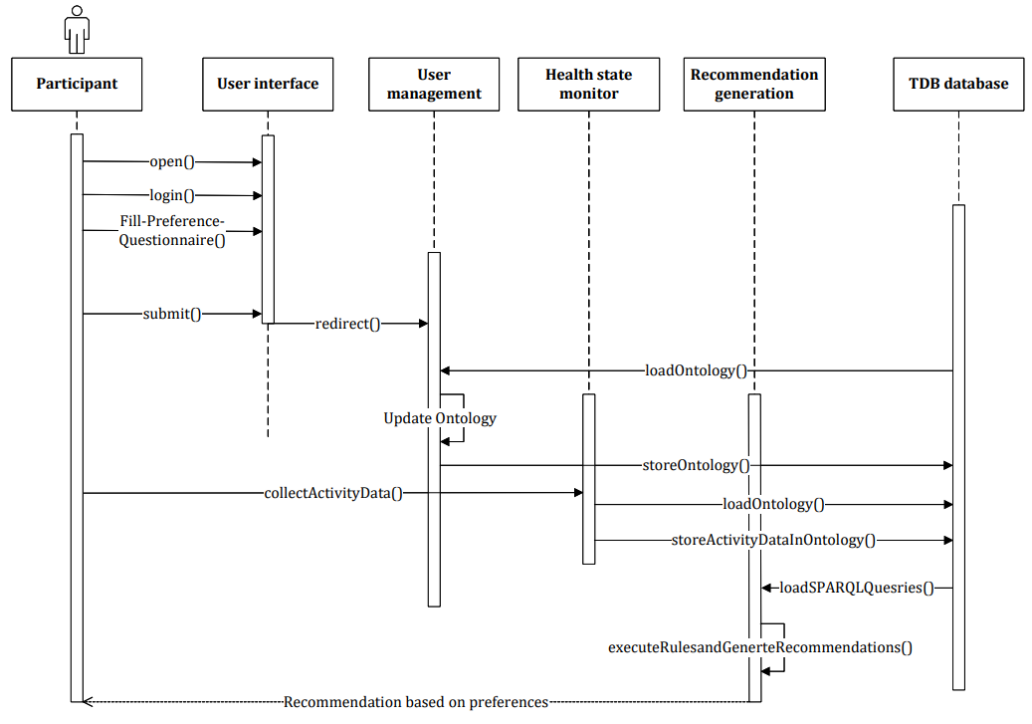
Unified Modeling Language sequence diagram for personalized recommendation generation and delivery. SPARQL: SPARQL Protocol and Resource Description Framework Query Language; TDB: tuple database.

Rules were composed of cause (A) and effect (B) to imply A → B. For each of the conditions mentioned in Table S3 in Multimedia Appendix 3, the recommendation module performed a SPARQL query every day to determine the type of recommended message to be delivered to each participant, as shown in the Unified Modeling Language sequence diagram (Figure 7). The execution of each of the predefined semantic rules specified in Table S3 in Multimedia Appendix 3 depended on the performance of the SPARQL queries, and the rules were created according to clinical guidelines [48-50]. This study subdivided 12 semantic rules into activity-level classification (n=10, 83%), weather classification (n=1, 8%), and satisfiability (n=1, 8%). The added concepts and rules were relatively easy to follow and use.

Observable and measurable parameters related to the activities and context of the individual participants on the time stamp were obtained based on SPARQL queries at preference-based intervals. The rules 1 to 8 in Table S3 in Multimedia Appendix 3 assigned truth values to variables to ensure consistency. We confirmed with HermiT that the correct recommendation message was triggered for specific situations. However, it was necessary to ensure that no variable combination makes the entire formula unsatisfiable; that is, no model can satisfy the process. We confirmed that only 1 message was triggered at a time. In this study, we had a formal guarantee that 2 *once a day* messages cannot be triggered simultaneously and there cannot be a model output by HermiT every time for every possible variable combination. If we put the different variables used in the first 10 rules (Table S3 in Multimedia Appendix 3) into the propositional variables (Table S1 in Multimedia Appendix 1), we will have an exponential number of *possible participants*.

As 2 messages cannot be triggered simultaneously to meet the exact requirements, we added a rule (rule 11), and the variable used in the proposal starts *once a day*. If rule 11 is false, the entire ruleset (deemed as significant conjunction) will be set to false, and then, there will be no model as output, and we will be able to *debug* our rules if needed. If it is set to true, we will have a formal guarantee that regardless of the true value we put in the rule base, 2 *once a day* messages will not be triggered at the same time.

### Ethics Approval

We have used simulated data for this study. Therefore, participants’ data have not been recorded or disclosed.

## Results

An e-coach system can use the messages presented in this study (Table S1 in Multimedia Appendix 1) to improve individual activities with proper goal management. Therefore, the e-coach system must access these messages stored in a KB during tailored recommendation generation. Both the asserted and inferred knowledge obtained through the reasoning method will be helpful to determine the most appropriate message.

The TDB database, as shown in Figure 7, was used as a KB in this study. The test used to verify the performance and reliability of the proposed *OntoRecoModel* ontology included SPARQL queries and a rule base. In ontology verification, we generated personalized and contextual activity recommendations according to the semantic rules to improve the individual’s physical activity to meet their activity goals. We executed all the semantic rules described in Table S3 in Multimedia Appendix 3 and used the Jena ARQ engine to run relevant SPARQL queries on the simulated data for the 8 test cases described in Table S4 in Multimedia Appendix 4. This helped to determine the type of recommendation message that would be generated, and we have presented our findings (rule-based recommendation generation for different cases) in Table 2. Several individual SPARQL queries are provided in Textbox S1 in Multimedia Appendix 6 as examples, and their results need to be combined to generate personalized recommendations to meet the e-coaching requirements. We achieved 100% precision in executing SPARQL queries to retrieve the necessary data.

Table 2 shows that participants can receive multiple motivational recommendation messages under ToDo and informal categories. The purpose of the e-coaching is to motivate participants (with motivational recommendation messages) for activities on day *n+1* based on the activity progression on day *n*, so that they can meet their weekly activity goals (*generic* or *personalized*) and maintain a healthy lifestyle. Proposition variable A-15 and A-16 (Table S1 in Multimedia Appendix 1) were the determinant of the weekly goal achievement and the delivery of the corresponding recommendation messages.

**Table 2:** Recommendation generation for different cases on day n for day n+1 (n>0).

Case	Activity status on day *n*	Recommendations for day *n+1*	
		ToDo	Informal
1	Goal achieved	A^a^-3, A-6, A-8, A-10, and A-12	A-13 and C^b^-1
2	Goal partially achieved	A-2, A-5, A-8, A-10, and A-11	A-14 and C-1
3	Goal partially achieved	A-1, A-5, A-7, A-9, and A-12	A-14 and C-1
4	Goal not achieved	A-1, A-5, A-7, A-9, and A-11	A-14 and C-1
5	Goal achieved	A-4, A-6, A-8, A-10, and A-12	A-13 and C-1
6	Goal partially achieved	A-4, A-5, A-8, A-9, and A-11	A-14 and C-1
7	Goal partially achieved	A-3, A-5, A-7, A-9, and A-12	A-14 and C-1
8	Goal not achieved	A-3, A-5, A-7, A-9, and A-11	A-14 and C-1

^a^A: activity recommendations.

^b^C: contextual recommendations.

## Discussion

### Principal Findings

The recommendation generation module used SPARQL queries and a rule base to generate personalized and contextual activity recommendations. There is no *false positive* situation based on the proposed ontology. According to the test cases in Table S4 in Multimedia Appendix 4, case 1 and case 5 achieved the daily activity goal; case 2, case 3, case 6, and case 7 achieved partial daily activity goal; and case 4 and case 8 ultimately failed to attain the daily activity goal. After combining the results of SPARQL queries with semantic rules, the related recommendation messages were updated, as shown in Table 2. The average execution time for all the SPARQL queries was between 0.1 and 0.3 seconds. The semantic rules described in Table S3 in Multimedia Appendix 3 represent the logic behind personalized recommendation message generation. The rule-based binary reasoning (if → 1, else → 0) helps to interpret the reason behind the delivery of a personal recommendation message.

The reasoning time of the proposed ontology was measured against the following reasoners available in Protégé: HermiT, Pellet, FaCT++, RacerPro, and KAON2; the corresponding processing times are shown in Table 3. The HermiT reasoner performed the best without reporting any inconsistencies.

**Table 3:** Comparative performance analysis of different reasoners available in Protégé.

Reasoner	Approximate reasoning time (seconds)
HermiT	2-3
Pellet	4-5
FaCT++	5-6
RacerPro	4-5
KAON2	5-6

The reading time after loading the ontology into the Jena workspace was approximately 1 to 2.5 seconds, with the *OWL_MEM_MICRO_RULE_INF* ontology specification (OWL full) in the *Terse RDF Triple Language* format, *in-memory* storage, and *optimized rule-based reasoner OWL rules*. Then, we used the Jena framework to query the ontology classes, predicates, subjects, and individuals in <1, <0.3, <0.4, and <2 seconds, respectively. Each ontology model (complete RDF diagram) was associated with a document manager (default global document manager: *OntDocumentManager*) to assist in processing ontology documents. All classes that represent the value of the ontology in the ontology API had *OntResource* as a general superclass with attributes (version information, comment, label, seeAlso, isDefinedBy, sameAs, and differentFrom) and methods (add, set, list, get, update, and delete). We implemented the RDF interface provided by Jena to maintain the modeled ontology and its instances in the TDB and load them back for further processing. Jena Fuseki was tightly integrated with TDB to provide a robust transactional persistent storage layer.

### Limitations and Future Scope

As explained in this study, we conducted the overall experiment on simulated data in a modeled e-coaching environment. This concept must be tested after integrating with a real-time activity e-coaching system, in which actual participants will be involved. Here, the personalized recommendation generation is rule-driven and straightforward. In Figure 2, the health state monitor and prediction module can be upgraded using data-driven ML approaches, followed by annotation of prediction results into the ontology. However, it is the future scope of this study.

In our conceptualized activity e-coaching, the recommendation generation module successfully searched the KB of motivational recommendation messages based on the rules in addition to the SPARQL results. The recommendation messages can be further personalized based on human behavior, liking for sports (eg, soccer), and the concept of reward bank. The components of the activity-related message can be further divided into indoor, outdoor, morning, afternoon, evening, and night activities. If a person has a dog and the e-coach system is aware of it, its recommendation generation module may suggest some activity recommendations involving the dog.

Table 2 shows that a participant can receive >1 recommendation message. It may lead to a message overloading problem. In future research, the recommendation process can be automated with ML algorithms (eg, time series and regression model) to select an optimal set of recommendations from feasible recommendations. The scope of the proposed ontology can be enhanced by conducting a study on a cluster of trials.

### Conclusions

This study created the *OntoRecoModel* ontology to generate and model personalized recommendation messages for physical activity coaching. The proposed ontology not only semantically annotates recommendation messages, their intention, and components but also models personal preference data, individual activity data, and contextual weather information (required for personalized recommendation generation). Moreover, we successfully verified the use of the proposed ontology in rule-based recommendation generation using the SPARQL query engine. This study also showed a direction to categorize recommendation messages according to the defined ontology rules. Furthermore, reasoning has helped to organize the recommendation messages into multiple aspects. The recommendation message categorization, their semantic annotation, and the ontological SPARQL queries enable the recommendation generation module to generate them based on preferences, activity data, and contextual weather data.

The *OntoRecoModel* ontology uses the OWL-based web language to represent the collected data in the RDF triple storage format. The performance of the proposed ontology was evaluated using simulated data from 8 test cases. The structure and logical consistency of the proposed ontology were evaluated using the HermiT reasoner. In future studies, we will recruit actual participants following the inclusion and exclusion criteria to replicate the entire test scenario and assess the effectiveness of the recommendation generation plan for goal evaluation.

## Appendix

Multimedia Appendix 1 Propositional variables and corresponding recommendation messages.

Multimedia Appendix 2 Different data types used in this study and their nature.

Multimedia Appendix 3 In-context recommendation conditions and corresponding rules (rule base) for test setup.

Multimedia Appendix 4 Description of the test cases.

Multimedia Appendix 5 Description of the contextual weather data.

Multimedia Appendix 6 Selected list of SPARQL Protocol and Resource Description Framework Query Language queries used in this study.

## Abbreviations

API: application programming interface
CDSS: clinical decision support system
KB: knowledge base
LPA: low physical activity
ML: machine learning
MPA: medium physical activity
OWL: Web Ontology Language
OWL-DL: Web Ontology Language–Description Logic
RDF: Resource Description Framework
SPARQL: SPARQL Protocol and Resource Description Framework Query Language
SSN: semantic sensor network
SWRL: Semantic Web Rule Language
TDB: tuple database
UiAeHo: University of Agder eHealth Ontology
VPA: vigorous physical activity
