# Supplementary material for: Personalized Recommendations for Physical Activity e-Coaching (OntoRecoModel): Ontological Modeling
Source: JMIR Med Inform. 2022 Jun 23;10(6):e33847. doi: 10.2196/33847 (PMC9282669; doi:10.2196/33847)
Supplement: Multimedia Appendix 1 [file medinform_v10i6e33847_app1.docx]

**Table S1.** Propositional variables and corresponding recommendation messages [49-52].

| Type | Propositional variable | Recommendation messages |
| --- | --- | --- |
| A-1 | Sedentary | Please continue light activity (e.g., sports 1-3 days/week, a walking goal of 5,000 to 7,499 steps/ day). |
| A-2 | Low_physically_active | Please continue more activity (e.g., sports 3-5 days/week, a walking goal of 7,500 to 9,999 steps/ day). |
| A-3 | Moderate_physically_active | Please continue same or more activity based on your goal (e.g., sports 3-5 days/week, a walking goal of 10,000 to 12,499 steps/ day). |
| A-4 | Vigorous_physically_active | Please continue same or more activity based on your goal (e.g., sports 5+ days/week, a walking goal of 12,500+ steps/ day). |
| A-5 | Sedentary_hour_negative | Please be active for z hr. more as today you were z hr. more sedentary beyond your goal. |
| A-6 | Sedentary_hour_positive | You were very active today and z hr. less sedentary; therefore, you can take that hr. of rest tomorrow. |
| A-7 | Steps_negative | Please continue x steps more tomorrow to achieve your weekly goal of x1 steps. |
| A-8 | Steps_positive | You have performed extra x steps today beyond your goal; therefore, you can do x steps less tomorrow or you can carry out same pace. You are x1 steps behind to achieve your weekly goal (OR) congratulations! You have achieved your weekly target. |
| A-9 | Activity_minute_negative | Please continue more activity of n minutes tomorrow to achieve n1 mins. of weekly goal. |
| A-10 | Activity_minute_positive | You have performed extra m minutes of activity today beyond your goal; therefore, you can be m mins. of less highly active tomorrow or you can carry out same pace. You are n1 mins. behind to achieve your weekly goal (OR) congratulations! You have achieved your weekly target. |
| A-11 | Insufficient_sleep | Please sleep 7-9 hrs./day (e.g., 10 pm. – 6 am or 11 pm. – 7 am). |
| A-12 | Sufficient_sleep | You had a nice sleep last night. Please keep it up. |
| A-13 | Daily_Goal_achieved | Good work. Please keep it up tomorrow. You are active and completed the goal for today.  **Overview:**  You have performed x steps today.  You slept y hrs.  You were sedentary for z hrs.  You were m minutes of medium active.  You were n minutes of highly active. |
| A-14 | Daily_Goal_not_achieved | You must improve to meet the daily goal. Please stay active tomorrow.  **Overview:**  You have performed x steps today.  You slept y hrs.  You were sedentary for z hrs.  You were m minutes of medium active.  You were n minutes of highly active. |
| A-15 | Weekly_Goal_achieved | Good work. Please keep it up next week. You are active and completed the goal for this week. |
| A-16 | Weekly_Goal_not_achieved | You must improve to meet the weekly goal. Please stay active next week and try to overcome the shortcomings of this week. |
| C-1 | Good_weather | Tomorrow the weather is favorable for outdoor activities. You can go for a walk or running. |
| C-2 | Bad_weather | Tomorrow the weather is not good for outdoor activities. Please plan indoor activities (e.g., walk or run-on treadmill) |
