# Supplementary material for: Personalized Recommendations for Physical Activity e-Coaching (OntoRecoModel): Ontological Modeling
Source: JMIR Med Inform. 2022 Jun 23;10(6):e33847. doi: 10.2196/33847 (PMC9282669; doi:10.2196/33847)
Supplement: Multimedia Appendix 2 [file medinform_v10i6e33847_app2.docx]

**Table S2.** different data types used in this study and their nature.

| Data type | Nature | Annotated data |
| --- | --- | --- |
| Activity data | Sensory data | Timestamp, steps, low physical activity (LPA), medium physical activity (MPA), vigorous physical activity (VPA), sedentary time |
| Contextual data | Sensory data | Timestamp, city, country, weather code, status, description, temp, real_feel, pressure, humidity, visibility, wind_speed |
| Goal data | Questionnaire-based preference data | Generic (e.g., system defined) or personalized |
| Response data | Questionnaire-based preference data | Recommendation data for activity |
| Interaction data | Questionnaire-based preference data | Mode (e.g., style, graph), frequency (e.g., hourly, quarterly, twice a day, daily, bi-weekly, weekly, monthly), medium (e.g., text) |
