# Supplementary material for: Personalized Recommendations for Physical Activity e-Coaching (OntoRecoModel): Ontological Modeling
Source: JMIR Med Inform. 2022 Jun 23;10(6):e33847. doi: 10.2196/33847 (PMC9282669; doi:10.2196/33847)
Supplement: Multimedia Appendix 3 [file medinform_v10i6e33847_app3.docx]

**Table S3.** In context recommendation conditions, and corresponding rules (rule-base) for test set-up.

| No. | Semantic Rule(s) [If] and Condition [Implies] |
| --- | --- |
| 1 | (hasSteps < 5000) AND (hasMPAMinutes < 30 OR hasVPAMinutes*2 < 30) AND (hasSedentaryBouts > 9*60) IMPLIES (Sedentary AND hasPhysicalActivityType) |
| 2 | (hasSteps < 7500) AND (hasMPAMinutes < 30 OR hasVPAMinutes*2 < 30) AND (hasSedentaryBouts < 9*60) IMPLIES (Low_physically_active AND hasPhysicalActivityType) |
| 3 | (hasSteps < 10000) AND (hasMPAMinutes > 30 OR hasVPAMinutes*2 > 30) AND (hasSedentaryBouts < 9*60) IMPLIES (Moderate_physically_active AND hasPhysicalActivityType) |
| 4 | (hasSteps >= 12500) AND (hasMPAMinutes > 30 OR hasVPAMinutes*2 > 30) AND (hasSedentaryBouts < 9*60) IMPLIES (Vigorous_physically_active AND hasPhysicalActivityType) |
| 5 | ((hasSedentaryBouts – daily_sedentary_goal_time as set in goal) > 0) IMLPIES (Sedentary_hour_negative)  ((hasSedentaryBouts – daily_sedentary_goal_time as set in goal) <= 0) IMLPIES (Sedentary_hour_positive) |
| 6 | ((hasSteps – daily_step_goal as set in goal) => 0) IMLPIES (Steps_positive)  ((hasSteps – daily_step_goal as set in goal) < 0) IMLPIES (Steps_negative) |
| 7 | ((hasMPAMinutes – daily_MPA_goal as set in goal) OR (hasVPAMinutes*2 – daily_VPA_goal as set in goal) => 0) IMLPIES (Activity_minute_positive)  ((hasMPAMinutes – daily_MPA_goal as set in goal) OR (hasVPAMinutes*2 – daily_VPA_goal as set in goal) < 0) IMLPIES (Activity_minute_negative) |
| 8 | (hasTotalSleepTime < (7*60) OR (hasTotalSleepTime < (daily_sleep_goal as set in goal *60)) IMPLIES (Insufficient_sleep)  (hasTotalSleepTime in_the_range_of (7*60 – 9*60) OR (hasTotalSleepTime in_the_range_of (daily_sleep_goal as set in goal *60)) IMPLIES (Sufficient_sleep) |
|  | ((hasSteps – daily_step_goal as set in goal) => 0) AND ((hasMPAMinutes – daily_MPA_goal as set in goal) OR (hasVPAMinutes*2 – daily_VPA_goal as set in goal) => 0) AND (hasTotalSleepTime => (daily_sleep_goal as set in goal *60)) AND ((hasSedentaryBouts – daily_sedentary_goal_time as set in goal) <= 0) IMLPIES (Daily_Goal_achieved) |
| 10 | ((hasSteps – weekly_step_goal as set in goal) => 0) AND ((hasMPAMinutes – weekly_MPA_goal as set in goal) OR (hasVPAMinutes*2 – weekly_VPA_goal as set in goal) => 0) AND (hasTotalSleepTime => (weekly_sleep_goal as set in goal *60)) AND ((hasSedentaryBouts – weekly_sedentary_goal_time as set in goal) <= 0) IMLPIES (Weekly_Goal_achieved) |
| 11 | (hasDescription == “clear sky” OR hasDescription == “few clouds” OR hasDescription == “scattered clouds”) IMPLIES (Good_weather)  (hasDescription == “broken clouds” OR hasDescription == “shower rain” OR hasDescription == “rain” OR hasDescription == “thunderstorm” OR hasDescription == “snow” OR hasDescription == “mist”) IMPLIES (Bad_weather) |
| 12 | (Sedentary + Low_physically_active + Moderate_physically_active + Vigorous_physically_active + Sedentary_hour_negative + Sedentary_hour_positive + Steps_negative + Steps_positive + Activity_minute_negative + Activity_minute_positive + Insufficient_sleep + Sufficient_sleep + Daily_Goal_achieved + Daily_Goal_not_achieved + Weekly_Goal_achieved + Weekly_Goal_not_achieved + Good_weather + Bad_weather = 1) |
