# Supplementary material for: Personalized Recommendations for Physical Activity e-Coaching (OntoRecoModel): Ontological Modeling
Source: JMIR Med Inform. 2022 Jun 23;10(6):e33847. doi: 10.2196/33847 (PMC9282669; doi:10.2196/33847)
Supplement: Multimedia Appendix 4 [file medinform_v10i6e33847_app4.docx]

**Table S4.** Description of the test cases.

| Case(s) | Activity data on day-n | Preference data | daily goal data^a^ | Weekly goal data |
| --- | --- | --- | --- | --- |
| Case-1 | Steps = 9200, MPA = 32.5, VPA = 6.0, sleep = 7.5 hrs., sedentary = 7.1 hrs. | Goal Type = “Generic”, Response Type = “Steps, Activity level, Sedentary bouts, Sleep time”, Interaction_mode = “Graph”, Interaction frequency = “Daily”, Interaction_medium = “Text” (e.g., pop-up notification) | Steps = 8,000, MPA = 30 – 43 mins. or VPA = 15 - 21.5 mins., sleep = 7 – 8 hrs., sedentary bouts = 8 - 9 hrs. without sleep time | Steps = 64,000, MPA = 210 – 300 mins. or VPA = 105 -150 mins., sleep = 49 - 56 hrs., sedentary bouts = 56 – 63 hrs. without sleep time |
| Case-2 | Steps = 8300, MPA = 15.5, VPA = 3.0, sleep = 6.5 hrs., sedentary = 9.1 hrs. | Goal Type = “Generic”, Response Type = “Steps, Activity level, Sedentary bouts, Sleep time”, Interaction_mode = “Graph”, Interaction frequency = “Daily”, Interaction_medium = “Text” (e.g., pop-up notification) | Steps = 8,000, MPA = 30 – 43 mins. or VPA = 15 - 21.5 mins., sleep = 7 – 8 hrs., sedentary bouts = 8 - 9 hrs. without sleep time | Steps = 64,000, MPA = 210 – 300 mins. or VPA = 105 -150 mins., sleep = 49 - 56 hrs., sedentary bouts = 56 – 63 hrs. without sleep time |
| Case-3 | Steps = 7300, MPA = 15.0, VPA = 3.5, sleep = 7.5 hrs., sedentary = 11.1 hrs. | Goal Type = “Generic”, Response Type = “Steps, Activity level, Sedentary bouts, Sleep time”, Interaction_mode = “Graph”, Interaction frequency = “Daily”, Interaction_medium = “Text” (e.g., pop-up notification) | Steps = 8,000, MPA = 30 – 43 mins. or VPA = 15 - 21.5 mins., sleep = 7 – 8 hrs., sedentary bouts = 8 - 9 hrs. without sleep time | Steps = 64,000, MPA = 210 – 300 mins. or VPA = 105 -150 mins., sleep = 49 - 56 hrs., sedentary bouts = 56 – 63 hrs. without sleep time |
| Case-4 | Steps = 7100, MPA = 14.5, VPA = 3.2, sleep = 6.5 hrs., sedentary = 11.6 hrs. | Goal Type = “Generic”, Response Type = “Steps, Activity level, Sedentary bouts, Sleep time”, Interaction_mode = “Graph”, Interaction frequency = “Daily”, Interaction_medium = “Text” (e.g., pop-up notification) | Steps = 8,000, MPA = 30 – 43 mins. or VPA = 15 - 21.5 mins., sleep = 7 – 8 hrs., sedentary bouts = 8 - 9 hrs. without sleep time | Steps = 64,000, MPA = 210 – 300 mins. or VPA = 105 -150 mins., sleep = 49 - 56 hrs., sedentary bouts = 56 – 63 hrs. without sleep time |
| Case-5 | Steps = 13500, MPA = 35, VPA = 10.5, sleep = 8.0 hrs., sedentary = 8.2 hrs. | Goal Type = “Personalized”, Response Type = “Steps, Activity level, Sedentary bouts, Sleep time”, Interaction_mode = “Graph”, Interaction frequency = “Daily”, Interaction_medium = “Text” (e.g., pop-up notification) | Steps = 12,000, MPA = 43 – 64 mins. or VPA = 21.5 - 43 mins., sleep = 7 - 8 hrs., sedentary bouts = <8 hrs. without sleep time | Steps = 84,000, MPA = 300 – 450 mins. or VPA = 150 - 300 mins., sleep = 49 - 56 hrs., sedentary bouts = <56 hrs. without sleep time |
| Case-6 | Steps = 13200, MPA = 31, VPA = 2.5, sleep = 6.5 hrs., sedentary = 9.8 hrs. | Goal Type = “Personalized”, Response Type = “Steps, Activity level, Sedentary bouts, Sleep time”, Interaction_mode = “Graph”, Interaction frequency = “Daily”, Interaction_medium = “Text” (e.g., pop-up notification) | Steps = 12,000, MPA = 43 – 64 mins. or VPA = 21.5 - 43 mins., sleep = 7 - 8 hrs., sedentary bouts = <8 hrs. without sleep time | Steps = 84,000, MPA = 300 – 450 mins. or VPA = 150 - 300 mins., sleep = 49 - 56 hrs., sedentary bouts = <56 hrs. without sleep time |
| Case-7 | Steps = 11200, MPA = 27, VPA = 1.5, sleep = 7.0 hrs., sedentary = 10.2 hrs. | Goal Type = “Personalized”, Response Type = “Steps, Activity level, Sedentary bouts, Sleep time”, Interaction_mode = “Graph”, Interaction frequency = “Daily”, Interaction_medium = “Text” (e.g., pop-up notification) | Steps = 12,000, MPA = 43 – 64 mins. or VPA = 21.5 - 43 mins., sleep = 7 - 8 hrs., sedentary bouts = <8 hrs. without sleep time | Steps = 84,000, MPA = 300 – 450 mins. or VPA = 150 - 300 mins., sleep = 49 - 56 hrs., sedentary bouts = <56 hrs. without sleep time |
| Case-8 | Steps = 11300, MPA = 29, VPA = 3.1, sleep = 6.0 hrs., sedentary = 9.1 hrs. | Goal Type = “Personalized”, Response Type = “Steps, Activity level, Sedentary bouts, Sleep time”, Interaction_mode = “Graph”, Interaction frequency = “Daily”, Interaction_medium = “Text” (e.g., pop-up notification) | Steps = 12,000, MPA = 43 – 64 mins. or VPA = 21.5 - 43 mins., sleep = 7 - 8 hrs., sedentary bouts = <8 hrs. without sleep time | Steps = 84,000, MPA = 300 – 450 mins. or VPA = 150 - 300 mins., sleep = 49 - 56 hrs., sedentary bouts = <56 hrs. without sleep time |

^a^MPA = 2*VPA
