# Supplementary material for: Personalized Recommendations for Physical Activity e-Coaching (OntoRecoModel): Ontological Modeling
Source: JMIR Med Inform. 2022 Jun 23;10(6):e33847. doi: 10.2196/33847 (PMC9282669; doi:10.2196/33847)
Supplement: Multimedia Appendix 5 [file medinform_v10i6e33847_app5.docx]

**Table S5.** Description of the contextual weather data.

| Data type | Day | Value |
| --- | --- | --- |
| Contextual data | Day-n (n > 0) | City = “Grimstad”, country = “NO”, weather code = 804, status = “Clouds”, description = “overcast clouds”, temp = “21.5”, real_feel = “22.0”, pressure =1003, humidity = 61, visibility = 10000, wind_speed = 3.32 |
|  | Day-n+1 (n > 0) | City = “Grimstad”, country = “NO”, weather code = 800, status = “clear”, description = “clear sky”, temp = 20.1, real_feel = 20.5, pressure = 1024, humidity = 73, visibility = 10000, wind_speed = 3.6. |
