# Supplementary material for: Personalized Recommendations for Physical Activity e-Coaching (OntoRecoModel): Ontological Modeling
Source: JMIR Med Inform. 2022 Jun 23;10(6):e33847. doi: 10.2196/33847 (PMC9282669; doi:10.2196/33847)
Supplement: Multimedia Appendix 6 [file medinform_v10i6e33847_app6.docx]

**Textbox S1.** Selected list of SPARQL queries used in this study.

| **Prefixes:**  PREFIX rdf: <http://www.w3.org/1999/02/22-rdf-syntax-ns#>  PREFIX owl: <http://www.w3.org/2002/07/owl#>  PREFIX rdfs: <http://www.w3.org/2000/01/rdf-schema#>  PREFIX xsd: <http://www.w3.org/2001/XMLSchema#>  PREFIX ssn: <http://purl.oclc.org/NET/ssnx/ssn#>  PREFIX: <http://www.co-ode.org/ontologies/uia/ont.owl#>  PREFIX status: http://www.w3.org/2003/06/sw-vocab-status/ns#  **Queries:**  **Rule for finding activity related parameters from an individual participant:**  SELECT ?participant ?datetime ?MPAMinutes ?SedentaryBouts ?steps ?totalsleeptime ?VPAMinutes ?PhysicalActivityType  WHERE {  ?participant :hasHealthRecord ?participanthealthrecord .  ?activitydata :hasBeenCollectedBy ?activitydatavalue .  ?activitydatavalue :hasSteps ?steps .  ?activitydatavalue : hasMPAMinutes ?hasMPAMinutes .  ?activitydatavalue : hasSedentaryBouts ?SedentaryBouts .  ?activitydatavalue : totalsleeptime ?totalsleeptime .  ?activitydatavalue : hasVPAMinutes ?VPAMinutes .  ?activitydatavalue : hasPhysicalActivityType ?PhysicalActivityType .  ?activitydatavalue :hasTimeStamp ?temporalentity .  ?temporalentity :hasDateTime ?datetime .  }  ORDER BY DESC (?datetime)  **Rule for finding contextual weather data for a day:**  SELECT ?participant ?datetime ?city ?country ?description ?humidity ?pressure ?realfeel ?status ?temp ?visibility ?weathercode ?windspeed  WHERE {  ?participant :hasContextData ?contextdata.  ?contextdata :hasBeenCollectedBy ?contextdatavalue .  ?contextdatavalue :hasCity ?city .  ?contextdatavalue : hasCountry ?country .  ?contextdatavalue : hasDescription ?description .  ?contextdatavalue : hasHumidity ?humidity .  ?contextdatavalue : hasPressure ?pressure .  ?contextdatavalue : hasRealFeel ?realfeel .  ?contextdatavalue : hasStatus ?status .  ?contextdatavalue : hasTemp ?temp .  ?contextdatavalue : hasVisibility ?visibility .  ?contextdatavalue : hasWeatherCode ?weathercode .  ?contextdatavalue : hasWindSpeed ?windspeed .  ?contextdatavalue :hasTimeStamp ?temporalentity .  ?temporalentity :hasDateTime ?datetime .  }  ORDER BY DESC (?datetime) |
| --- |
